# Quantitative Evaluation of the Association Between Fixation Stability and Phoria During Short-Term Binocular Viewing

**DOI:** 10.3389/fnins.2022.721665

**Published:** 2022-03-10

**Authors:** Sang-Yeob Kim, Byeong-Yeon Moon, Hyun Gug Cho, Dong-Sik Yu

**Affiliations:** Department of Optometry, Kangwon National University, Samcheok, South Korea

**Keywords:** fixation stability, bivariate contour ellipse areas (BCEA), phoria, binocular vision, eye tracker

## Abstract

**Purpose:**

Fixation stability for binocular anomalies with a phoria cannot be detected by direct observations. This study aimed to quantitatively evaluate fixation stability using an eye tracker rather than direct directions in binocular vision with abnormal and normal phorias.

**Methods:**

Thirty-five and 25 participants with abnormal and normal phoria, respectively, were included in the study. The horizontal and vertical gaze points and convergence were recorded for 10 s using a remote eye tracker while binocularly viewing a target on a display screen 550 mm away. Fixation stability was quantified using bivariate contour ellipse areas (BCEA).

**Results:**

The fixation stability for all participants-based evaluations as a single cluster in the abnormal phoria group was lower than that in the normal phoria group (*p* = 0.005). There was no difference between the two groups in the evaluation based on the BCEA for each participant-based evaluation (*p* = 0.66). Fixation stability was also more related to convergence for the abnormal phoria group than for the normal phoria group (*r* = 0.769, *p* < 0.001; *r* = 0.417, *p* = 0.038, respectively).

**Conclusion:**

As the first study to evaluate fixation stability using an eye-tracker to differentiate between abnormal and normal phoria for non-strabismus, these findings may provide evidence for improving the evaluation of binocular vision not detected with clinical diagnostic tests.

## Introduction

Fixation stability is the ability of the eyes to hold the image of an object on the fovea by keeping a constant steady gaze on the fixation target. The ability to maintain steady fixation plays important roles in eye movements, including saccadic and pursuit functions (Subramanian et al., 2013; Hessels et al., 2018). These eye movements are widely researched in ophthalmology, neurology, psychology, cognitive science, and human factors (Holmqvist et al., 2011; Laretzaki et al., 2011; Metsing and Ferreira, 2016; Krauzlis et al., 2017; Thielen et al., 2019). Eye movement anomalies associated with visual organic or functional anomalies, particularly fixation stability, may be present in amblyopia, nystagmus, maculopathy, myasthenia gravis, glaucoma, strabismus, and non-strabismic binocular vision disorders (Subramanian et al., 2013; Otero-Millan et al., 2014; Mihara et al., 2017; Tarita-Nistor et al., 2017; Ghasia et al., 2018; Montesano et al., 2018).

In clinical practice, for evaluating fixation stability in binocular function, an objective assessment with observable eye movements is carried out to determine whether visual fixations are normal or abnormal. For normal binocular function, both eyes should be able to sustain precise fixation for 10 s (Scheiman and Wick, 2014). Even if the eye is evaluated as being able to normally fixate in the practical test, the eye may not be completely still; i.e., there are types of micro-movements present, including microsaccades, tremors, and drifts (Holmqvist et al., 2011). Diagnostic tests for evaluating fixation stability in clinical practice are quick and easy to use and include the Northeastern State University College of Optometry (NSUCO) oculomotor test and the Southern California College of Optometry (SCCO) 4 + system (Metsing and Ferreira, 2016). However, micro-movements are not observable by these tests without special equipment such as eye trackers and micro-perimeters (van der Lans et al., 2011; Hirasawa et al., 2018). Although fixation stability can be measured by eye trackers clinically other than direct observation under the binocular vision, most of them were evaluated under manifest strabismus. Additionally, an objective assessment or diagnostic test for fixation stability by observation with the naked eye is not detected in non-strabismus; therefore, it is evaluated as normal. Thus, evaluation of fixation stability in clinical practice is limited to visual observable manifests of strabismus with or without amblyopia.

Phoria is an eye condition for two visual axes of the eyes not to be directed toward the point of fixation, in the absence of fusion. Thus, phoria can be detected by a dissociated state such as the von Graefe’s test, the Maddox rod test, the cover test, and observation of fixation in clinical tests (Schroeder et al., 1996). However, phoria for non-strabismic binocular anomalies with fusion such as convergence insufficiency, convergence excess, divergence insufficiency, divergence excess, basic exophoria, and basic esophoria (Cooper et al., 2010) can’t be detected by clinical tests during binocular viewing. A quantitative analysis of eye movements using an infrared tracking system during the cover test was studied (Barnard and Thomson, 1995), but the study was conducted under a dissociated state rather than during binocular viewing. Therefore, eye movements using eye-tracking systems, i.e., an objective assessment of fixation stability, could be a new clinical test evaluating the differences between phorias.

Various studies on fixation stability related to binocular functions using an eye tracker or a perimeter have been reported. These include the quantitative analysis of eye movements in the cover test, methods quantifying eye stability using the bivariate contour ellipse area (BCEA; Castet and Crossland, 2012), binocular coordination of each of the types of fixation eye movements (Otero-Millan et al., 2014), the relationship between visual acuity and fixation stability (Chung et al., 2015), and the impact of refractive error on eye fixation (Wahl et al., 2019). Variations in the fixation of abnormal binocular functions have mostly been reported in strabismus. Multiple studies have used eye trackers to report fixation stability during monocular and binocular viewing (Economides et al., 2016; Chen et al., 2018). However, no study has quantitatively analyzed fixation stability in phoria not detected by diagnostic tests in clinical practice, i.e., for non-strabismus binocular vision. Phoria involves latent deviation of both visual axes from the orthoposition that requires vergence in order for bifixation to be maintained. Therefore, we anticipate that the fixation stability for abnormal and normal phoria will differ in horizontal and vertical gaze positions and in convergence due to differences in stress on the visual sensory and oculomotor systems for maintaining a clear and single binocular vision.

The purpose of this study was to quantitatively evaluate the fixation stability of abnormal and normal phoria during binocular viewing. Using a display screen, fixation stability was evaluated for horizontal and vertical eye positions and for convergence. This study was designed as a pilot study that applied an eye tracker to tests of binocular vision. To achieve this, fixation stability was measured non-invasively during short-term (10 s) binocular viewing in free space. The viewing was done using a remote (screen-based) eye tracker (based on the eye gaze) at a near distance under conditions similar to the diagnostic tests in clinical practice.

## Materials and Methods

### Experimental Protocol

**FIGURE 1:**
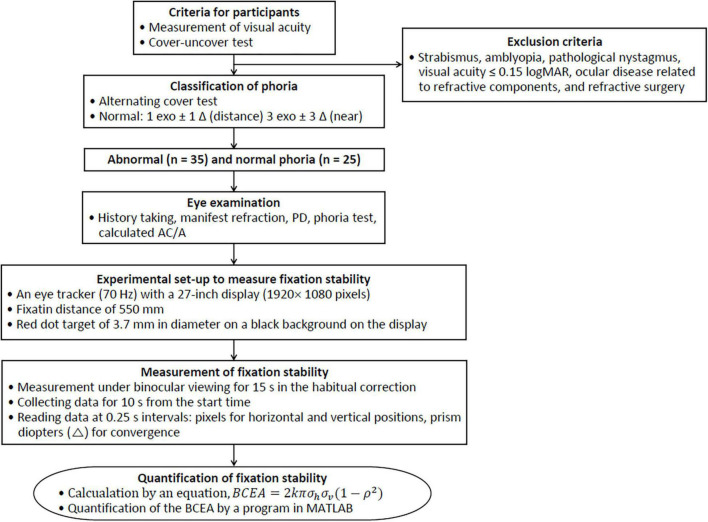
Flow chart for the quantitative evaluation of fixation stability.

As shown in Figure 1, the experimental procedures consisted of classification of abnormal and normal phoria, preliminary eye examination, measurement of fixation stability using an eye tracker, and quantitative evaluation of the fixation stability.

### Participants

Participants were classified into abnormal and normal (as control) groups based on phoria to quantitatively evaluate fixation stability. The binocular status for consecutive participants attending an eye examination was obtained using the cover-uncover test after the measurement of visual acuity. Participants with distance (6 m) or near (40 cm) tropia were excluded, while those with non-strabismic binocular vision anomalies were included in this study. Further exclusion criteria were strabismus, amblyopia, pathological nystagmus, poor vision under 0.15 logMAR, ocular disease related to refractive components, and refractive surgery. All the tests, including tests for phoria, were performed with habitual spectacles, contact lenses, or non-spectacles without supplemental examinations for the current prescriptions. Phoria was measured using the alternating cover test with prism bars. Negative and positive values represent exophoria (exo) and esophoria (eso), respectively. Irrespective of symptoms and the ranges of vergences, the normal criteria for distance and near phoria are 1 exo ± 1 Δ and 3 exo ± 3 Δ, respectively (Wajuihian, 2019). The participants included 35 university students with abnormal phoria and 25 with normal phoria. Types of the abnormal phoria group were classified based on phoria and the calculated AC/A (accommodative convergence to accommodation ratio) according to Scheiman and Wick’s study (Darko-Takyi et al., 2016).

This study was approved by the Institutional Review Board of Kangwon National University (KWNUIRB-2018-10-002-001) and was conducted in accordance with the tenets of the Declaration of Helsinki. All participants provided written informed consent.

### Eye Examination

All the participants underwent ocular examinations, including history-taking (to obtain information about the chief ocular complaints), assessment of manifest refraction using a phoropter (VT-SE; Topcon, Tokyo, Japan), and assessments using visual charts (ACP-8; Topcon, Tokyo, Japan). These examinations were to determine the corrected or uncorrected visual acuity for habitual spectacle or non-spectacle wearers. Other examinations included measurement of refractive power for habitual spectacle wearers using a lensmeter (LM-15; Topcon, Tokyo, Japan) and inter-pupillary distance (PD) using a PD meter (PD-5; Topcon, Tokyo, Japan). The phoria test was performed using prism bars (HB 16; Astron International, Naples, FL, United States) while the calculated AC/A ratio was the sum of the PD (cm) and the difference in phoria between the near and distance phoria divided by 2.50 diopters (D).

### Experimental Set-Up

Fixation stability was assessed using the Clinical Eye Tracker (Version 18.04; Thomson Software Solutions, Hatfield, United Kingdom) system equipped with a non-invasive and measurable remote (screen-based) eye tracker (Tobii eyeX; Tobii Technology, Stockholm, Sweden). This eye-tracking system, with a frequency of 70 Hz, operating distance of 50–90 cm, and available screen size of 27 inches, records the direction of eye movements such as horizontal gaze position (x-axis), vertical gaze position (y-axis), and convergence such as over-convergence (eso) or under-convergence (exo) based on the gaze point, by asking both eyes to constantly look at the target in the center on the screen. Fixation data were recorded with three infrared (IR) LEDs and two IR video cameras. This system is also capable of extracting the eye position information through most spectacle prescriptions with higher-powered lenses and operates under a wide range of ambient light conditions (Thomson, 2017). The performance of the eye tracker used in this study is sufficient for some classes of research applications and can be employed to measure fixation parameters (Gibaldi et al., 2017).

**FIGURE 2:**
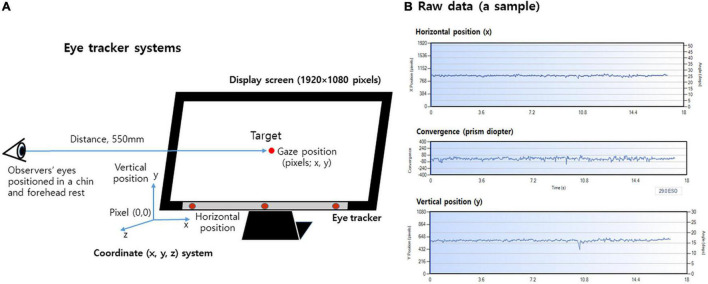
Experimental set-up of the display and eye tracker coordinate systems. **(A)** Eye tracker coordinate systems and display screen for measuring fixation stability. **(B)** Sample of the raw data for the horizontal gaze position (x-axis), vertical gaze position (y-axis), and convergence.

The experimental set-up of the display and eye tracker coordinate systems are shown in Figure 2. The distance between the center of the screen [tilt angle of 15 degrees (deg)] and the eye was 550 mm and the screen size was 27 inches (1920 × 1080 pixels). Although this system automatically compensates for head movements under natural viewing conditions, the participants sat using the chin and forehead rest to avoid head movement effects during the evaluation of fixation stability. Before measuring the gaze position, system calibration was performed according to the user manual instructions provided on the system, similar to how the participant looks at the dots in the four corners of the screen until the dots explode.

The target for the fixation stability testing was the more stable central fixation target rather than the pericentral fixation targets. In this study, the central fixation target that was designed for use in evaluating fixation stability was a red dot target 3.7 mm (12 pixels) in diameter on a black background. This target has a size that corresponds to a visual acuity of −0.66 logMAR (visual angle of 0.385 deg) at 550 mm The gaze center of both eyes was directed toward the target in the center of the screen.

### Measurement Procedures

After the eye examination and experimental set-up, the gaze positions were measured during binocular viewing of a target at 550 mm under normal room illumination. The eye-tracking system used in this study can measure fixation stability during binocular viewing under natural viewing conditions worn with spectacles or contact lenses. The measurements were conducted on participants with or without corrected visual acuity of equal or better than 0.15 logMAR, without the current prescriptions. The reason for measuring both habitual wearers and non-wearers is because their fixation stability or visual performance might be affected, in addition to experiencing blurry or clear vision through uncorrected or currently corrected refractive errors.

Before the measurements, participants were instructed to fixate on the target that was presented for 15 s after automatically starting the viewing along with the cash register sounds as a sound of the start of the measurement procedure. They were asked not to move their head during the binocular viewing. The measurements were carried out after simulating the measurement process. Gaze positions for evaluating fixation stability were measured in both eyes by default in this eye tracker system. According to the system user manual, the binocular gaze positions are calculated from the average gaze positions of the right and left eyes. These data are generally less noisy than the monocular data and are used for the general eye position analysis during fixation. Although the recorded data were obtained for 15 s, the data that were used were for 10 s from the start time, which is the same time as that of the evaluation for fixation stability in clinical practice. The recorded data were read at 0.25 s intervals. However, the data were collected within the ± 0.02 s range based on a close and + direction priority to the measurement time when these data were affected by blinking or noise. For example, if there were no data at 2.75 s, then the priorities were in the order of 2.76, 2.74, 2.77, and 2.73 s. Recorded data were read as pixel data for the x- and y-axes (horizontal and vertical positions), and the pixels were converted to deg units using a conversion factor of 0.032 deg/pixel as needed. Convergence in fixation stability was also measured to evaluate the changes in the near vergence of both eyes. For the analysis, the collected data were changed by placing the zero point in the target position (x: 960, y: 540 pixels) to offset the values.

Quantification of the fixation stability can be determined by calculating the area of an ellipse which encompasses fixation points for a given probability (P%) of eye positions during fixation. The calculated area is the BCEA. Therefore, a smaller BCEA value is indicative of greater fixation stability, whereas a larger value is unstable. The BCEA can be calculated using the following equation (Eq. 1) (Timberlake et al., 2005; Pirdankar and Das, 2016; Wahl et al., 2019).


(1) $$B\,C\,E\,A=2\,k\,\pi \,\sigma_{h}\,\sigma_{v}\,\left(1-\rho^{2}\right)$$

where σ_*h*_ and σ_*v*_ are the standard deviations (SD) of the gaze points in the horizontal and vertical positions, respectively; ρ is the product-moment correction of the two positional components; and k is dependent upon the probability area chosen (P) (Eq. 2).


(2) $$P=1-e^{-k}$$

where e is the base of the natural logarithm. Therefore, k is 1.146 for 68.2% (± 1SD) and 3.079 for 95.4% (± 2SD). The fixation data were then analyzed using custom MATLAB programs that were used to calculate the BCEA (see Supplementary Material); the ellipse area that contains the probability area of the fixation points was shown. If BCEA values were not normally distributed, the BCEA values were converted into their logarithms for statistical analysis.

### Data Analysis

All the data collected were statistically analyzed using MedCalc (Version 12.7.7.0; MedCalc Software, Ostend, Belgium). The normality of distribution was verified by the Kolmogorov–Smirnov test. BCEA (deg^2^) was normalized by logarithmic transformation when necessary. Independent samples *t*-test for normality and Mann-Whitney U test for non-normality were used to compare the mean values of the abnormal and normal phoria groups. Pearson’s correlation coefficient (r) was used to assess correlations. A *p*-value of ≤ 0.05 was considered statistically significant.

## Results

### Participants Characteristics

Table 1 shows the demographic and clinical characteristics of the participants in this study. Significant differences were found between the abnormal (*n* = 35) and normal phoria groups (*n* = 25) in the distance and near phoria, and in the calculated accommodative convergence to accommodation ratio (calculated AC/A) (independent samples *t*-test, *t* = 2.93, *p* = 0.006; *t* = 2.98, *p* = 0.005; *t* = 2.18, *p* = 0.03, respectively). In particular, those in the abnormal phoria group had characteristics of near binocular anomalies, including convergence insufficiency.

**TABLE 1:** Demographic and clinical characteristics of the participants.

Parameter	Abnormal phoria (*n* = 35)	Normal phoria (*n* = 25)	*p*-value
Gender (male/female, n)	17/18	14/11	
Age (years)	21.97 (2.04)	21.04 (1.62)	
**Visual acuity (logMAR)**			
Right and left eye	0.02 (0.04)	0.03 (0.05)	*t* = 0.51, 0.61
**Optical correction (D, diopters)**			
Spherical equivalent	−4.85 (2.50)	−5.03 (2.88)	*t* = 0.11, 0.91
Dioptric different between both eyes	−0.21 (0.77)	−0.29 (1.04)	
Ratio of correction (n)	26/35	17/25	
**Phoria (Δ, prism diopters)**			
Distance	−3.68 (5.33)	−1.00 (0.87)	*t* = 2.93, 0.006
Near	−8.46 (9.58)	−3.44 (2.31)	*t* = 2.98, 0.005
Calculated AC/A (Δ/D, prism/diopter)	4.44 (2.03)	5.30 (0.98)	*t* = 2.19, 0.03
**Binocular vision anomalies (n)**			
Normal		25	
Convergence insufficiency (CI)	18		
Convergence excess	8		
Basic exophoria	6		
Basic esophoria	3		

*Data are presented as the mean (standard deviation) and the number of participants. Minus and plus signs in phoria indicate exophoria and esophoria, respectively.*

*Binocular vision anomalies are based on the phoria and AC/A ratio.*

### Fixation Stability of Participants

**FIGURE 3:**
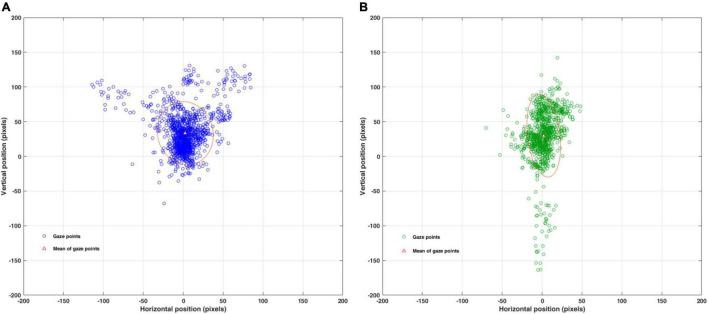
Quantification of the fixation stability for all participants using the BCEA, as assessed using MATLAB. BCEA: bivariate contour ellipse area; BCEA of 68.2% for ± 1SD. **(A)** BCEA (pixel^2^) fixation stability for abnormal phoria. **(B)** BCEA (pixel^2^) fixation stability for normal phoria. BCEA (pixel^2^) can be converted to BCEA (deg^2^) using a conversion factor of 0.032 deg/pixel.

Figure 3 shows the quantification of the fixation stability by BCEA using the MATLAB program (R2015b; The MathWorks, Natick, MA, United States) for all the participants as a single cluster. In the plot of the BCEA, although comparative statistics were not applicable because of the insufficient data, differences for descriptive statistics between the abnormal and normal phoria groups were 1.2 deg^2^ for 1SD and were greater in the abnormal group. These results are also shown in Table 2, which shows the fixation stability for the horizontal and vertical gaze points. BCEA for the abnormal and normal groups were 5.11 and 3.91 deg^2^ for 1SD. Descriptive statistics indicated distinct differences between the two groups, considering that there were differences (0.34–0.60 deg^2^) in a previous study of fixation stability for the strabismus and normal groups (Ghasia et al., 2018). In gaze point for each participant, BCEA for each of the abnormal and normal groups are 0.73 and 0.74 deg^2^ for 1SD. The accuracy of the fixation was calculated as the average pixels or angles between the measured fixation positions and the positions of the fixation targets. Differences in the accuracy of the vertical position and gaze angle between the abnormal and normal phoria groups existed in the collective data for all the participants (independent samples *t*-test, *t* = −2.32, *p* = 0.02; *t* = 2.85, *p* = 0.005, respectively), but not for each participant’s data (independent samples *t*-test, *t* = −0.50, *p* = 0.60; *t* = 0.44, *p* = 0.66, respectively).

**TABLE 2:** Gaze points and fixation stability during binocular viewing.

Parameter	Abnormal phoria	Normal phoria	*p*-value
**Gaze points for all the participants**			
Horizontal position (pixels)	2.52 (23.22)	2.00 (13.95)	*t* = −0.64, 0.52
95% CI for the mean	1.21 to 3.82	1.09 to 2.91	
Vertical position (pixels)	33.51 (30.07)	29.87 (39.46)	*t* = −2.32, 0.02
95% CI for the mean	31.82 to 35.20	27.30 to 32.44	
Gaze angle (deg)	1.28 (1.00)	1.39 (0.88)	*t* = 2.85, 0.005
95% CI for the mean	1.22 to 1.33	1.39 to 1.45	
BCEA (deg^2^) ± 1SD	5.11	3.91	
**Gaze points for each participant**			
Horizontal position (pixels)	2.20 (20.56)	1.81 (11.96)	*t* = −0.09, 0.93
Vertical position (pixels)	33.61 (28.20)	29.18 (37.13)	*t* = −0.50, 0.60
Gaze angle (deg)	1.29 (0.95)	1.39 (0.79)	*t* = 0.44, 0.66
BCEA (deg^2^) ± 1SD	0.73 (0.62)	0.74 (0.38)	*z* = 1.07, 0.28
Log (BCEA) (deg^2^) ± 1SD	−0.27 (0.35)	−0.20 (0.25)	*t* = 0.94, 0.35

*Data are presented as the mean (standard deviation). CI, confidence interval; Deg, degree; BCEA, bivariate contour ellipse area; SD, standard deviation.*

### Convergence Stability of Participants

**FIGURE 4:**
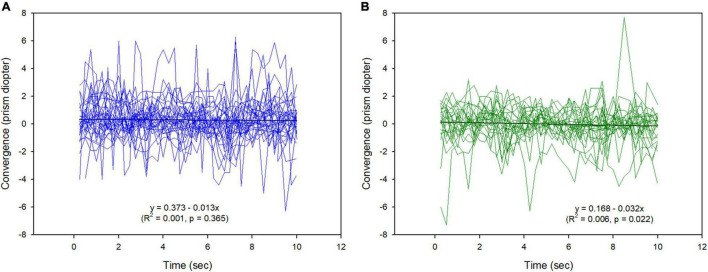
Changes in convergence during binocular viewing. **(A)** Abnormal phoria group. **(B)** Normal phoria group.

Figure 4 shows changes in convergence during short-term binocular viewing for all the participants. In the representation by linear equations, the change in convergence with time (during short-term) was not found in the abnormal group (*p* = 0.365), but was very weak and exo-shift in the normal group (*p* = 0.022). The range of change was wider in the abnormal group than in the normal group. Change in convergence was exo-shift over time (during short-term) in the normal phoria group, with a wider range of change in convergence in the abnormal phoria group than in the normal group. Table 3 shows the convergence stability during binocular viewing. Differences in the convergence between abnormal and normal phoria groups as the fluctuation in the vergence existed in the collective data of all the participants (independent samples *t*-test, *t* = −5.41, *p* < 0.001), with no difference in the evaluation based on each participant’s data.

**TABLE 3:** Convergence stability during binocular viewing.

	Abnormal phoria	Normal phoria	*p*-value
**For all the participants**			
Convergence (Δ)	0.31 (1.38)	0.002 (1.22)	*t* = −5.41, *p* < 0.001
95% CI for the mean	0.23 to 0.39	−0.08 to 0.08	
BCEA (sec × Δ) ± 1SD	28.53	24.89	
**For each participant**			
Convergence (Δ)	0.30 (0.62)	0.01 (0.70)	*t* = −1.71, 0.09
BCEA (sec × Δ) ± 1SD	20.91 (14.20)	18.66 (7.08)	*t* = −0.81, 0.42
Log BCEA (sec × Δ) ± 1SD	1.26 (0.22)	1.24 (0.16)	*t* = −0.33, 0.74

*Data are presented as the mean (standard deviation). SD, standard deviation; CI, confidence interval; BCEA, bivariate contour ellipse area.*

In comparing the BCEA for convergence between the abnormal and normal phoria groups, although comparative statistics were not applicable due to insufficient data, differences in descriptive statistics between the abnormal and normal phoria groups were 3.64 s × prism diopter (Δ) for 1SD and were greater in the abnormal group. However, these differences did not exist in the evaluation based on each participant’s data.

### Relation Between Fixation-Related Variables

Table 4 shows the relationship between the BCEA and fixation-related variables, including phoria, the AC/A ratio, and variables expressed as the SD in the gaze positions and convergence, to determine the factors of BCEA. For the abnormal phoria group, the direct relationship with the BCEA (i.e., fixation stability) was strongly positively correlated with the SDs of vertical and horizontal gazes, and with the SD of convergence (*r* = 0.877, 0.829, 0.769; *p* < 0.001 for all). In addition, similar results were obtained using the log(BCEA) instead of the BCEA, and the indirect relationship of fixation stability was also strongly positively correlated with convergence and the SD of the horizontal gaze. Other correlations revealed indirect relationships between the SDs of the horizontal and vertical gazes; between the SDs of convergence and distance phoria; and between the AC/A ratio, near phoria, and distance phoria. For the normal phoria group, the direct relationship with fixation stability was strongly positively correlated with the SD of the vertical gaze and moderately positively correlated with the SD of the horizontal gaze (*r* = 0.881, *p* < 0.001; 0.583, *p* = 0.002, respectively). In addition, similar results were observed using the log(BCEA) instead of the BCEA. The indirect relationship of fixation stability was also moderately positively correlated with convergence and the SD of the horizontal gaze (*r* = 0.642, *p* < 0.01). Other correlations showed no direct or indirect relationships between the variables.

**TABLE 4:** Pearson’s correlation coefficients between fixation-related variables.

Parameter	Abnormal phoria (*p*-value)	Normal phoria (*p*-value)
BCEA vs. vertical gaze	0.877 (*p* < 0.001)	0.881 (*p* < 0.001)
BCEA vs. horizontal gaze	0.829 (*p* < 0.001)	0.583 (*p* = 0.002)
BCEA vs. convergence	0.769 (*p* < 0.001)	0.417 (*p* = 0.038)
Horizontal gaze vs. convergence	0.947 (*p* < 0.001)	0.642 (*p* = 0.001)
Horizontal gaze vs. vertical gaze	0.560 (*p* < 0.001)	0.163 (*p* = 0.435)
Vertical gaze vs. convergence	0.539 (*p* = 0.001)	0.201 (*p* = 0.335)
Near phoria vs. distance phoria	0.881 (*p* < 0.001)	0.147 (*p* = 0.482)
Near phoria vs. AC/A	0.866 (*p* < 0.001)	0.898 (*p* < 0.001)
Distance phoria vs. AC/A	0.533 (*p* = 0.001)	−0.184 (*p* = 0.378)

*BCEA, bivariate contour ellipse area.*

## Discussion

In our study, fixation stability (as assessed using an eye tracker) was evaluated under similar diagnostic conditions, i.e., during binocular viewing for 10 s at a near distance for non-strabismus with abnormal phoria, which cannot be detected by general diagnostic tests for fixation stability (Scheiman and Wick, 2014). Our main findings for the abnormal phoria group compared to the normal phoria group were as follows: In the evaluation based on all the participants; the eye tracker could be used to apply and establish the fixation stability test for abnormal and normal phoria; accuracy, representing the distance from the fixation target to the gaze point, was lower in the abnormal group; the fixation stability for the BCEA determined by the SDs of the horizontal and vertical gaze points was also lower in the abnormal group, and convergence itself was larger in the abnormal group, and the convergence stability determined by the BCEA was lower in the abnormal group. In the evaluation based on each participant, there was no difference between the two groups. In addition, the values of the correlations were high, implying strong correlations of the variables with BCEA, either directly or indirectly.

The participants’ demographic and ocular characteristics were not significantly different, except for binocular vision anomalies and parameters related to phoria, such as distance phoria, near phoria, and the calculated AC/A. These findings clearly distinguished between the abnormal and normal phoria groups.

Gaze points during binocular viewing were found to differ in the analysis based on the horizontal and vertical positions as well as the visual angle. In the analysis for all the participants, the vertical position of 33.51 ± 30.07 pixels in the abnormal phoria group was larger than the 29.87 ± 39.46 pixels in the normal phoria group. However, the visual angle of 1.28 ± 1.00 deg in the abnormal phoria group was smaller than the 1.39 ± 0.88 deg in the normal phoria group, while those of the horizontal positions were similar. These values represent the accuracy, which is determined by the average difference between the target and the gaze positions (Holmqvist et al., 2012). Accuracy for the gaze points (based on the horizontal and vertical positions) and the visual angle were different between abnormal and normal phoria. These differences are due to the evaluation of the gaze points considering the direction in the horizontal and vertical positions, but not considering the direction in the visual angle (Bellmann et al., 2004; Cesareo et al., 2015). In the analysis for each participant, the horizontal and vertical positions and the visual angle showed no significant differences between the abnormal and normal phoria groups. The SDs of the horizontal and vertical gaze points and those of the visual angle during fixating for each participant were lower than the SDs for all the participants. These differences are due to individual-based data processing because each participant had the effect of SD reduction. Moreover, there were differences in the shapes of the BCEAs between the two groups. In a different case (75%) (Fujii et al., 2002) from our case (68.2% for 1 SD), the stability of the BCEA meant that at least 75% of all fixation points were within the 2 deg, and the unstability of the BCEA meant that at least 75% of all fixation points were within the 4 deg. However, it is necessary to discuss which of 75 or 68.2% is appropriate. In our study, the horizontal and vertical components were relatively comparable. The horizontal gaze points (as a component of the BCEA) in the participants with abnormal phoria were wider than those with normal phoria. From comparing two shapes, the horizontal gaze was evaluated as unstable in the abnormal group and stable in the normal group. The binocular viewing in the case of participants with abnormal phoria required greater vergence to compensate for the horizontal phoria (i.e., positive or negative fusional vergence for exo and eso, respectively) (Cooper et al., 2010), in order to maintain a clear and single image in participants with a high frequency of near phoria, particularly convergence insufficiency. For these reasons, abnormal phoria had less fixation stability than normal phoria.

In fixation stability based on the BCEA, BCEA ± 1SD for each participant in the abnormal and normal phoria groups were 0.73 and 0.74 deg^2^, respectively. In a study of healthy young participants, the BCEA value was 1.67 deg^2^ during standard automated perimetry (Hirasawa et al., 2018). In another study of 29 healthy participants, the BCEA reported using fundus-tracking perimetry was 0.61 deg^2^, while the value was 4.79 deg^2^ in patients with low vision (Amore et al., 2013; Cesareo et al., 2015). These results show that the values for both groups in our study were within the normal range, and binocular vision was possible during short-term binocular viewing. Fixation stability in the present study was moderate compared to that reported in previous similar studies. In the abnormal and normal phoria groups, the log(BCEA) ± 1SD for each participant were −0.27 and −0.20 deg^2^, respectively. Fixation stability in the present study was also moderate when compared to the −0.88, −0.48, and −0.24 deg^2^ that were reported for the control groups in previous studies (González et al., 2012; Raveendran et al., 2014; Shaikh et al., 2016). These differences may be related to differences in the eye-tracking systems or measuring instruments, sampling rates, fixation targets, and test durations between studies (Steinman, 1965; Amore et al., 2013; Raveendran et al., 2019).

Statistical comparisons were not applicable as *n* = 1, but the BCEA in the abnormal phoria group for all the participants based on descriptive statistics was larger than that in the normal phoria group. However, no difference was found in the comparison of the BCEA for each participant in the abnormal and normal phoria groups. The differences in the BCEA for all the participants and for each participant were statistically significant, which was based on the SD used to determine the BCEA in the horizontal and vertical positions (Timberlake et al., 2005; Amore et al., 2013). Fixation stability for strabismus and normal participants in a previous study were 0.64–0.90 deg^2^ and 0.30 deg^2^, respectively (Ghasia et al., 2018); showing distinct differences between the two groups. The eyes are not totally still during fixation but rather make continuous miniature movements, including microsaccades, drifts, and tremors which keep the retina in motion (Krauzlis et al., 2017). Thus, we anticipated that the eye movements would increase with increasing phoria, even in the absence of a tropia. However, there was no clear difference between the abnormal and normal phoria groups in our study. The lack of a clear difference in fixation stability means that the difference is smaller between the abnormal and normal phoria group with binocular vision than between strabismus without binocular vision and normal phoria with binocular vision groups. The lack of clear difference does not also mean that the fixation stability test for abnormal and normal phoria cannot be applied using the eye tracker. To verify a more distinct difference in fixation stability between abnormal and normal phoria, the status of the binocular vision that can deteriorate over time (such as long-term rather than short-term) (Amore et al., 2013), and larger targets rather than smaller targets (Steinman, 1965; Bellmann et al., 2004), may be needed. Since the eye movements directly affect early visual cortex activity, impaired visual acuity can reduce fixation stability (Thielen et al., 2019). However, the visual acuity for all the participants in this study was equal to or greater than 0.15 logMAR. Therefore, there was no effect on fixation stability due to impaired visual acuity.

To maintain binocular vision, individuals with abnormal phoria must use vergence (Darko-Takyi et al., 2016). Therefore, we expected that eye movements would continuously occur during binocular viewing, which would eventually affect the fixation stability. In our evaluation for all the participants, BCEA that was determined as a function of convergence and time, which was previously unknown, was larger and lower stability in abnormal phoria than in normal phoria. However, in the evaluation based on each participant, there were no statistically significant differences between the two groups, although the abnormal phoria group had numerically larger values than the normal phoria group. The mean phoria values at a near distance for the abnormal and normal phoria groups were −8.46 Δ and −3.44 Δ for exo, respectively. In the evaluation for all the participants, the mean convergence values to maintain binocular viewing were 0.31 Δ and 0.002 Δ for eso, respectively. Eso-shift during binocular viewing means that more convergence is required to compensate for more exo. This also means that the binocular accuracy for abnormal phoria is lower than that of normal phoria (Blakey et al., 2020). Statistical comparisons were not applicable, but convergence stability expressed by the BCEA in abnormal phoria for all the participants was larger than that in normal phoria. This result shows that the convergence stability is reduced due to the increase in more fusional vergence, which is required to maintain the binocular vision for the increased phoria (Cooper et al., 2010). In the evaluation of the convergence stability for each participant, no difference was found between abnormal and normal phoria. The convergence stability based on each participant was lower than that based on all participants. These results depend on the difference in the SDs between the two groups. To verify a more distinct difference in convergence stability between abnormal and normal phoria, as mentioned previously, in the evaluation of fixation stability, the status of the binocular vision that can deteriorate over time, such as in long-term and with larger targets, is also needed. Overall, our results demonstrate that binocular viewing in abnormal phoria requires greater vergence; for this reason, change takes place in convergence.

Correlations between variables related to fixation stability were analyzed. Variables that can affect the BCEA included phoria, the AC/A ratio related to phoria, the SD of the horizontal and vertical positions of the gaze points, and the convergence. As shown in Table 4, the main differences between the abnormal and normal phoria groups include the direct relationship with BCEA in the horizontal and vertical positions in both phoria groups; however, convergence was only related to the abnormal phoria. In the abnormal phoria group, phoria and the AC/A ratio had a weak indirect relationship, which was shown through convergence; however, in the normal phoria group, the indirect relationship was not shown. In the abnormal group, the direct and indirect relationships with BCEA are presumed to mean that several factors are involved in maintaining binocular vision due to the instability of the binocular system. Such an interpretation can be found in the characteristics (including reduced convergence and near deviation) in the abnormal phoria group consisting of convergence insufficiency, convergence excess, basic exophoria, and basic esophoria (Blum et al., 2015; Darko-Takyi et al., 2016; Yu et al., 2018). These vergence fluctuations may therefore lead to a reduction in binocular fixation stability.

Eye movements perceived by the unaided eye in cover test is 1 prism diopter to 2 prism diopters, i.e., 0.57 deg to1.14 deg (Ludvigh, 1949; Cantó-Cerdán et al., 2018). Therefore, fixation stability of less than these values are not observable by an objective assessment or diagnostic test without special equipment such as eye trackers and micro-perimeters. Nevertheless, the potential limitations of this study are the lack of diversity in the participants’ characteristics and experimental conditions, especially in binocular anomalies such as eso or hyperopia, longer fixation times, and various stimuli. In addition, the BCEA has limitations since it assumes that fixations are normally distributed in space. However, this is the first study to evaluate fixation stability using an eye-tracker to distinguish between abnormal and normal phoria for non-strabismus not detected by diagnostic tests. Therefore, this study could not be conducted under various conditions to exclude the variables. As discussed above, to establish a more distinct difference in fixation stability between abnormal and normal phoria groups, further investigations are required under conditions of deteriorated binocular vision. As it is possible to evaluate the fixation stability of the normal and the abnormal phoria by the eye-tracker, clinical application may be applicable to various phoria.

In summary, rather than using the diagnostic tests for fixation stability in clinical practice, we conducted a quantitative analysis using the BCEA (i.e., the fixation stability), including the horizontal and vertical gaze positions and convergence. Our evaluation based on all the participants showed that the stability of the abnormal phoria group was lower than that of the normal phoria group. There was no difference between the two groups in terms of the evaluation based on the BCEA for each participant. However, we found that fixation stability is related to convergence. When fixation stability cannot be detected using the clinical diagnostic tests in the evaluation of binocular anomalies with phoria such as non-strabismus, i.e., when a detailed examination is required, assessment of fixation stability using an eye tracker is recommended.

## Data Availability Statement

The original contributions presented in the study are included in the article/Supplementary Material, further inquiries can be directed to the corresponding author/s.

## Ethics Statement

This study was approved by the Institutional Review Board of Kangwon National University (IRB approval number: KWNUIRB-2018-10-002-001). The patients/participants provided their written informed consent to participate in this study.

## Author Contributions

S-YK and D-SY conceived and designed the study. S-YK collected the data and wrote the main manuscript text. D-SY performed supervision and project administration. B-YM and HC carried out data analyses and interpretation. All authors participated in the review of the manuscript.

## Conflict of Interest

The authors declare that the research was conducted in the absence of any commercial or financial relationships that could be construed as a potential conflict of interest.

## Publisher’s Note

All claims expressed in this article are solely those of the authors and do not necessarily represent those of their affiliated organizations, or those of the publisher, the editors and the reviewers. Any product that may be evaluated in this article, or claim that may be made by its manufacturer, is not guaranteed or endorsed by the publisher.
