# Supplementary material for: Quantitative Evaluation of the Association Between Fixation Stability and Phoria During Short-Term Binocular Viewing
Source: Front Neurosci. 2022 Mar 10;16:721665. doi: 10.3389/fnins.2022.721665 (PMC8965591; doi:10.3389/fnins.2022.721665)
Supplement: Supplementary file 2 [file Table_2.DOCX]

**Quantification of the BCEA by a program in MATLAB**

% Plotting probability ellipses of the bivariate normal distribution

clear all; close all; clc;

% Create data sets

Horizontal_Position = [x_1_, x_2_, x_3_, ‧‧‧x_n_];

Vertical_Position = [y_1_, y_2_, y_3_, ‧‧‧y_n_];

% Calculate movements of the data sets

% Observed

Horizontal_mean = mean(Horizontal_Position); % x Position mean

Vertical_mean= mean(Vertical_Position); % y Position mean

CV = cov(Horizontal_Position,Vertical_Position); % covariance of x and y

[Evec, Eval]=eig(CV); % Eigen values and vectors of covariance matrix

% Plot observed multivariate contours

% Observed data

xCenter = Horizontal_mean; % ellipses centered at sample averages

yCenter = Vertical_mean;

theta = 0 : 0.01 : 2*pi; % angles used for plotting ellipses

% compute angle for rotation of ellipse

% rotation angle will be angle between x axis and first eigenvector

x_vec= [1 ; 0]; % vector along x-axis

cosrotation =dot(x_vec,Evec(:,1))/(norm(x_vec)*norm(Evec(:,1)));

rotation =pi/2-acos(cosrotation); % rotation angle

R = [sin(rotation) cos(rotation); ...

-cos(rotation) sin(rotation)]; % create a rotation matrix

% create chi squared vector

chisq = [2.291 6.158]; % percentiles of chi^2 dist df=2

% size ellipses for each quantile

for i = 1:length(chisq)

% calculate the radius of the ellipse

xRadius(i)=(chisq(i)*Eval(1,1))^.5; % primary

yRadius(i)=(chisq(i)*Eval(2,2))^.5; % secondary

% lines for plotting ellipse

x{i} = xRadius(i)* cos(theta);

y{i} = yRadius(i) * sin(theta);

% rotate ellipse

rotated_Coords{i} = R*[x{i} ; y{i}];

% center ellipse

x_plot{i}=rotated_Coords{i}(1,:)'+xCenter;

y_plot{i}=rotated_Coords{i}(2,:)'+yCenter;

end

% Set up plot

figure

xlabel('Horizontal position (pixels)')

ylabel('Vertical position (pixels)')

hold on

% Plot Gaze points

plot(Horizontal_Position,Vertical_Position,'o');

% Plot contours

for j = 1:length(chisq)

plot(x_plot{j},y_plot{j})

end

% Plot Center point

scatter(mean(Horizontal_Position),mean(Vertical_Position),'red','^');

legend('Gaze points','68.2%', '95.4%', 'Center point')
